# PSYCHOLOGICAL WELL-BEING AND CHANGE OF COGNITIVE FUNCTION OVER 4 YEARS

**DOI:** 10.1093/geroni/igz038.3002

**Published:** 2019-11-08

**Authors:** Dexia Kong, Dexia Kong, XinQi Dong

**Affiliations:** 1 Rutgers University, New Brunswick, New Jersey, United States; 2 Rutgers University, The State University of New Jersey, New Brunswick, New Jersey, United States

## Abstract

This study aims to examine the relationship between psychological-wellbeing (including depressive symptoms, loneliness, anxiety, hopelessness, and stress) and cognitive function during a 4-year period among U.S. Chinese older adults. Data were from 2,300 PINE participants who completed in-home interviews at baseline, 2-year and 4-year follow-up. Cognitive function was assessed by five individual cognition tests at baseline and follow-up interviews. Mixed-effects regression models were conducted. A trend of decline in global cognition and multiple cognitive domains was observed. The study findings showed that depressive symptoms (Estimate= -0.03, p<0.001), anxiety (Estimate = -0.02, p<0.001), hopelessness (Estimate = -0.01, p<0.01), and stress (Estimate = -0.01, p<0.001) were associated with poorer global cognitive function at baseline. Baseline psychological well-being was not associated with changes in cognitive function during a 4-year period. Significant predictors of rate of change in cognitive function will be discussed. Potential explanations and implications of these findings will be presented.

